# *In Vivo* RNA Delivery to Hematopoietic Stem and Progenitor Cells *via* Targeted Lipid Nanoparticles

**DOI:** 10.1021/acs.nanolett.3c00304

**Published:** 2023-03-29

**Authors:** Dennis Shi, Sho Toyonaga, Daniel G. Anderson

**Affiliations:** † Department of Chemical Engineering, Massachusetts Institute of Technology, Cambridge, Massachusetts 02139, United States; ‡ David H. Koch Institute for Integrative Cancer Research, Massachusetts Institute of Technology, Cambridge, Massachusetts 02139, United States; § FUJIFILM Pharmaceuticals U.S.A., Inc., Cambridge, Massachusetts 02142, United States; ∥ Harvard-Massachusetts Institute of Technology, Division of Health Science and Technology, Massachusetts Institute of Technology, Cambridge, Massachusetts 02139, United States; ⊥ Institute for Medical Engineering and Science, Massachusetts Institute of Technology, Cambridge, Massachusetts 02139, United States

**Keywords:** lipid nanoparticle, RNA, hematopoietic stem cells, antibody targeting, blood disorders

## Abstract

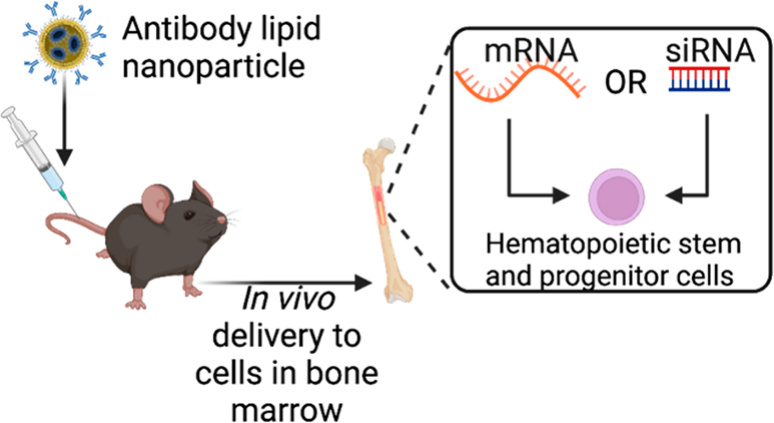

Ex vivo autologous hematopoietic stem cell (HSC) gene therapy has provided new therapies for the treatment of hematological disorders. However, these therapies have several limitations owing to the manufacturing complexities and toxicity resulting from required conditioning regimens. Here, we developed a c-kit (CD117) antibody-targeted lipid nanoparticle (LNP) that, following a single intravenous injection, can deliver RNA (both siRNA and mRNA) to HSCs *in vivo* in rodents. This targeted delivery system does not require stem cell harvest, culture, or mobilization of HSCs to facilitate delivery. We also show that delivery of Cre recombinase mRNA at a dose of 1 mg kg^–1^ can facilitate gene editing to almost all (∼90%) hematopoietic stem and progenitor cells (HSPCs) *in vivo*, and edited cells retain their stemness and functionality to generate high levels of edited mature immune cells.

Hematopoietic stem cells (HSCs) are rare cells residing in the bone marrow (about 1 in 10,000 bone marrow cells^1^) that are responsible for the generation and maintenance of the body’s immune system through hematopoiesis.^2^ These cell types, which are capable of self-renewal and multilineage differentiation through proximal progenitor cells, are attractive targets for gene therapy as genomic correction of the defective gene at the stem cell level results in corrected progeny and long-lasting therapeutic efficacy of many diseases.^3^ In recent years, clinical success with autologous stem cell gene therapy has been demonstrated for diseases such as β-globinopathies,^4^ primary immunodeficiencies,^5^ and metabolic disorders.^6^

In the process of *ex vivo* autologous gene therapy, the patient’s own stem cells are first harvested and then cultured *ex vivo* for gene transfer or editing before the edited hematopoietic stem and progenitor cells (HSPCs) are reinfused back into the patient.^7^ For successful engraftment of edited cells, the patient must undergo conditioning regimens to make space within the hematopoietic niche by depleting the existing cells using chemotherapeutic agents such as busulfan or radiotherapy.^8^ The intensity of the conditioning regimen is dependent on the number of corrected cells required for therapeutic effect.^8^ In diseases that require high intensity conditioning, patients often experience debilitating side effects such as infertility or off-target organ toxicity,^9^ which hinders the widespread applicability of *ex vivo* stem cell transplantation. In addition, due to the personalized patient-specific nature of autologous gene therapy, which requires specialized manufacturing centers, the cost of *ex vivo* gene therapy is often quite prohibitive for patients.^10^

To expand the therapeutic potential of HSC gene therapy to a broader set of patients, one alternative could be to direct *in vivo* delivery of RNA to HSCs within the patient’s body without the need for stem cell harvesting, culturing, and patient conditioning. Current reported *in vivo* delivery strategies of HSCs utilize helper-dependent adenoviruses which require mobilization for efficient transduction followed by selection of edited cells through low doses of chemotherapy.^11−13^ Use of a nonviral lipid nanoparticle (LNP) delivery system for HSCs has the potential to overcome the limitations of adenoviruses by enabling redosing and simplifying production. With the advancement of CRISPR Cas9-based base editors^14^ and prime editors^15^ that allow for targeted gene editing without inducing DNA double strand breaks, LNPs that can delivery RNA to HSCs *in vivo* may enable genetic editing therapies for various hematologic diseases.

Here, we develop an antibody conjugated lipid nanoparticle targeted to CD117 for *in vivo* delivery of both siRNA and mRNA to HSPCs. CD117, also known as c-Kit, is a canonical marker of many stem cell populations and is expressed on the surface of both human and mouse HSPCs.^16^ In addition, CD117 is rapidly internalized and has been used in the context of selective HSPC depletion *via* CD117 targeted antibody drug conjugates.^17,18^ We hypothesized that the conjugation of this antibody to lipid nanoparticles could allow for receptor-mediated delivery of RNA into HSPCs *in vivo*. We first evaluated the utility of anti-CD117 targeted LNPs using different lipid combinations to deliver RNA to a hematopoietic progenitor cell line *in vitro*. This was followed by modification of different formulation parameters to facilitate the *in vivo* delivery of siRNA targeting CD45. We then evaluated the potential of our optimized formulation in a transgenic Ai14 mouse model as a surrogate model for gene editing. A single intravenous administration of anti-CD117-LNP at a dose of 1 mg kg^–1^ Cre mRNA achieved ∼90% conversion to TdTomato^+^ cells in both HSPCs and long-term hematopoietic stem cells (LT-HSCs). In addition, by monitoring the level of TdTomato^+^ cells in the peripheral blood, we also demonstrated that transfected HSPCs generated high levels of edited progeny. Fourteen weeks after LNP injection, we observed 90% TdTom^+^ myeloid cells, 70% TdTom^+^ B cells, and about 50% TdTom^+^ T cells as well as near 100% TdTom^+^ erythrocytes.

To allow for conjugation of HSPC targeting antibodies, a functionalized maleimide PEG-lipid was incorporated during nanoparticle formulation that reacted *via* thiol-maleimide chemistry with free thiols generated from partially reduced anti-CD117 (clone 2B8). Successful conjugation was determined by eluting the LNPs *via* size exclusion chromatography (Figure S1) as well as *via* DLS which showed a size increase of about 20 nm after conjugation (Figure 1a). Polydispersity index of the LNPs remained narrow (<0.2) after conjugation and RNA encapsulation efficiency remained unchanged (Table S1).

**Figure 1:**
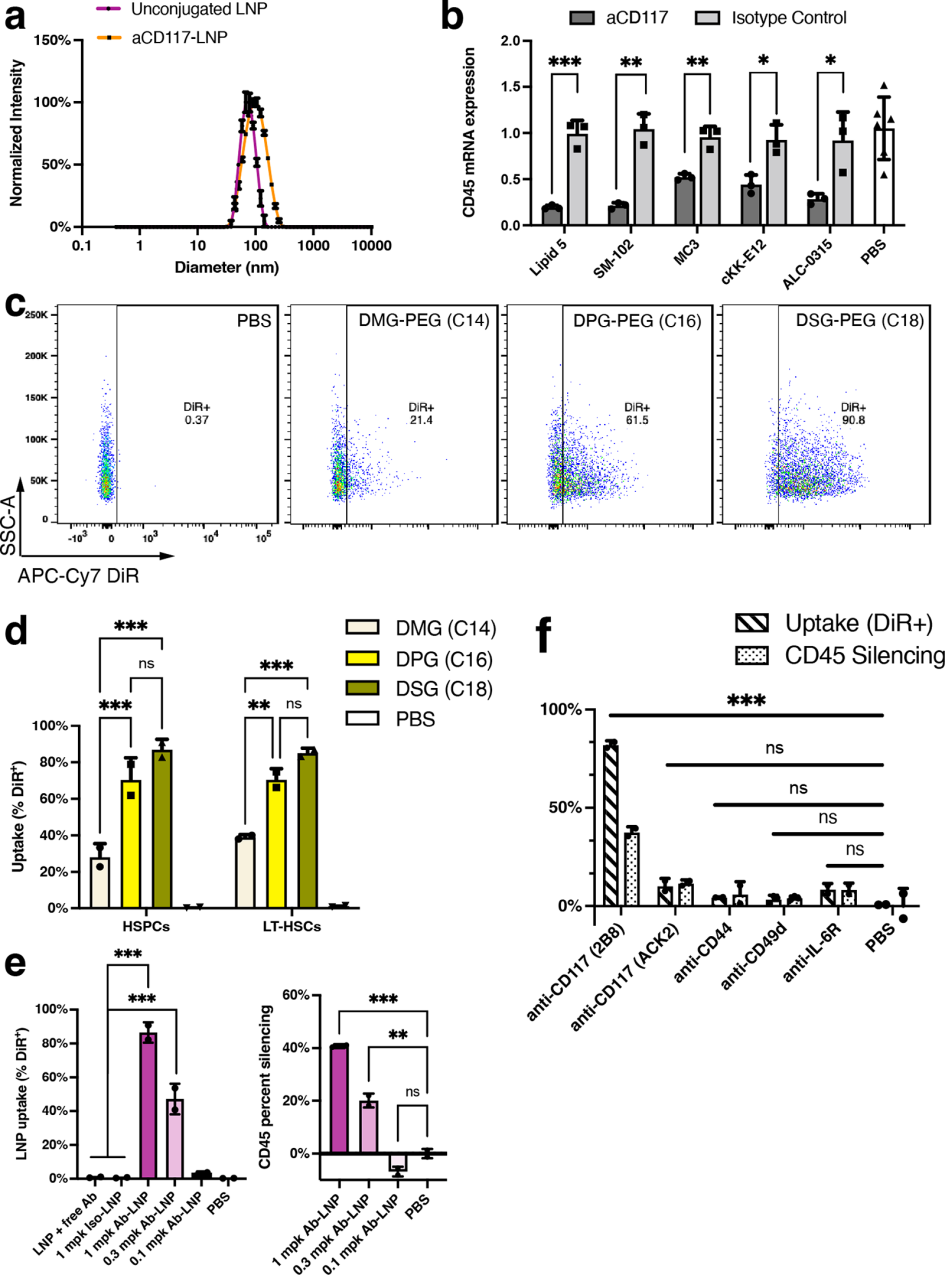
Anti-CD117 LNPs can deliver siCD45 both *in vitro* and *in vivo*. (a) DLS of unconjugated LNP *vs* conjugated LNP. (b) Anti-CD117 LNPs (dosed at 50 ng siCD45 per well) showed potent knockdown *in vitro* in EML cell line (*n* = 3 wells). Statistics were performed with multiple unpaired two-tailed *t* tests comparing the isotype control of each group. (c) Representative FACS scatter plots for the DiR signal in bone marrow HSPCs for different PEG-lipids at 1 mg kg^–1^ siCD45. (d) Quantification of the level of LNP uptake in HSPCs and LT-HSCs. LNPs were administered at a dose of 1 mg kg^–1^ siCD45 (*n* = 2 mice, initial screening, two-way ANOVA with Tukey’s multiple comparison test). (e) Dose–response of anti-CD117 LNP using the C18-PEG lipid. Bone marrow LSK cells were analyzed 72 h after LNP administration for uptake (left) and functional CD45 knockdown (right) (*n* = 2 mice, screening, one-way ANOVA with Tukey’s multiple comparison test). (f) Screening of alternate markers for *in vivo* HSPC delivery at a dose of 1 mg kg^–1^ siCD45 (*n* = 3 mice for anti-CD49d condition, *n* = 2 mice for all other conditions, one-way ANOVA with Dunnett’s multiple comparison test). Data are represented as mean ± SD (*P < 0.05, **P < 0.01, ***P < 0.001).

For initial evaluation and screening of formulation parameters for optimal HSPC delivery, we encapsulated an siRNA against CD45 as a method to assess functional RNA delivery to HSPCs in a cell specific and cost-effective manner. To confirm that formulated anti-CD117 LNPs were amenable for RNA delivery based on our initial hypothesis, we first evaluated these Ab-LNPs *in vitro* in the murine EML (CD117^+^ hematopoietic progenitor) cell line.^19^ We formulated targeted Ab-LNPs with various published potent ionizable lipids (DLin-MC3-DMA, cKK-E12, Moderna’s Lipid 5, SM-102, and ALC-0315) with siCD45 and assessed RNA level knockdown with qPCR 48 h after transfection. All tested formulations showed very high level of silencing (50–80%) *in vitro* at a dose of 50 ng siRNA while LNPs conjugated to the isotype control were not effective at transfecting these cells (Figure 1b).

Next, after *in vitro* validation, we evaluated the efficacy of delivery to HSPCs *in vivo*. For *in vivo* applications, we decided to use ALC-0315 as the ionizable lipid for formulations because ALC-0315 has been FDA approved for its use in the Pfizer/BioNTech COVID mRNA vaccine with demonstrated safety and tolerability. It has previously been reported that increasing the circulation time of nanoparticles (via modulation of the PEG-lipid component^20^) increases delivery efficacy to the bone marrow.^21−23^ Therefore, we investigated the effect of the length of the PEG-lipid alkyl chain (C14, C16, or C18) on Ab-LNP uptake in bone marrow HSPCs *in vivo*. Ab-LNP formulations with different PEG-lipids were fluorescently labeled with lipid dye DiR and then administered intravenously. Then 72 h after administration, bone marrow cells were collected and analyzed for nanoparticle uptake in HSPCs (defined as Lin^–^ Sca1^+^ c-Kit^+^) and LT-HSCs (defined as Lin^–^ Sca1^+^ c-Kit^+^ CD34^–^ CD135^–^) *via* flow cytometry (Figure 1c,d, gating strategy shown in Figure S2). The level of LNP uptake greatly increased when PEG-lipids with longer alkyl chains, which have slower desorption from the LNP due to increased hydrophobic interactions, were incorporated—going from around 30% uptake with conventional C14-PEG lipid to almost 90% uptake with C18-PEG lipid. This improvement in delivery with increased alkyl chain length of the PEG-lipid was also demonstrated with mRNA Ab-LNP formulations (Figure S3). We also explored the ligand density of antibodies on the nanoparticle surface by varying the amount of antibody used during the conjugation reaction and found an optimum ligand density for LNP uptake (Figure S4). Using the optimized formulation, we then evaluated the dose-dependent response in anti-CD117 LNP uptake and functional knockdown of CD45 at doses of 0.1, 0.3, and 1 mg kg^–1^ siRNA (2, 6, and 20 μg of RNA, respectively). No uptake and silencing were observed at 0.1 mg kg^–1^ siRNA, while the highest dose corresponded to about 40% CD45 knockdown (Figure 1e). In addition, covalent conjugation of the antibody, rather than just passive adsorption, was shown to be essential for antibody mediated delivery, as LNPs mixed with free antibody showed minimal levels of uptake along with the isotype control LNP.

We also wanted to investigate the use of other antibodies that could potentially be used for *in vivo* delivery to HSPCs. We chose a small panel of other receptors that are expressed on HSPCs (CD49d, CD44, and IL-6R) and conjugated LNPs with antibodies to those receptors. In addition, we also investigated another clone of CD117 (clone ACK2). Among the targets screened, only CD117 was effective for LNP uptake and RNA delivery (Figure 1f). The other antibodies might not be suitable for targeted delivery to HSPCs due to factors such as the clone of the antibody used or receptor dependent factors such as expression level, internalization rate, or off-target tissue expression levels that could serve as antigen sinks. Interestingly, CD117 demonstrated a clonal difference in the performance of Ab-LNPs indicating that the choice of antibody against a specific cell target greatly affects its utility for targeted delivery using antibody decorated lipid nanoparticles. Clone 2B8 is a nonantagonistic clone while clone ACK2 is reported to be antagonistic;^17^ however, no depletion of bone marrow HSPCs was observed following Ab-LNP administration with either clone or with Ab-LNPs conjugated to any of the other antibodies (Figure S5). In the context of anti-CD117 ADCs, it has been shown that while both 2B8 and ACK2 are efficiently internalized *in vitro*, ADCs using clone 2B8 were much better at depleting HSCs.^17^ Currently, the reason for this difference in antibody clones is unknown, and further work will be performed to evaluate whether there are certain principles that govern the suitability of some antibody clones over others for their use in targeted delivery systems.

Having optimized the formulation for maximal uptake and delivery of siRNA, next we transitioned to evaluating the delivery efficacy of anti-CD117 LNPs with mRNA. For this, we utilized the transgenic Ai14 mouse model, which contains a LoxP-flanked stop cassette that prevents the transcription of fluorescent protein TdTomato.^24^ Upon Cre recombination (via delivery of Cre mRNA), the stop cassette is excised out, and the cell then constitutively expresses TdTomato (Figure 2a). The level of gene editing in HSPCs following treatment with our Ab-LNP was then determined using flow cytometry. In addition, after LNP administration, we tracked the mature immune cells in the peripheral blood long-term to see the level of TdTomato expression in edited progeny at 2, 4, 6, 8, and 14 weeks (Figure 2b).

**Figure 2:**
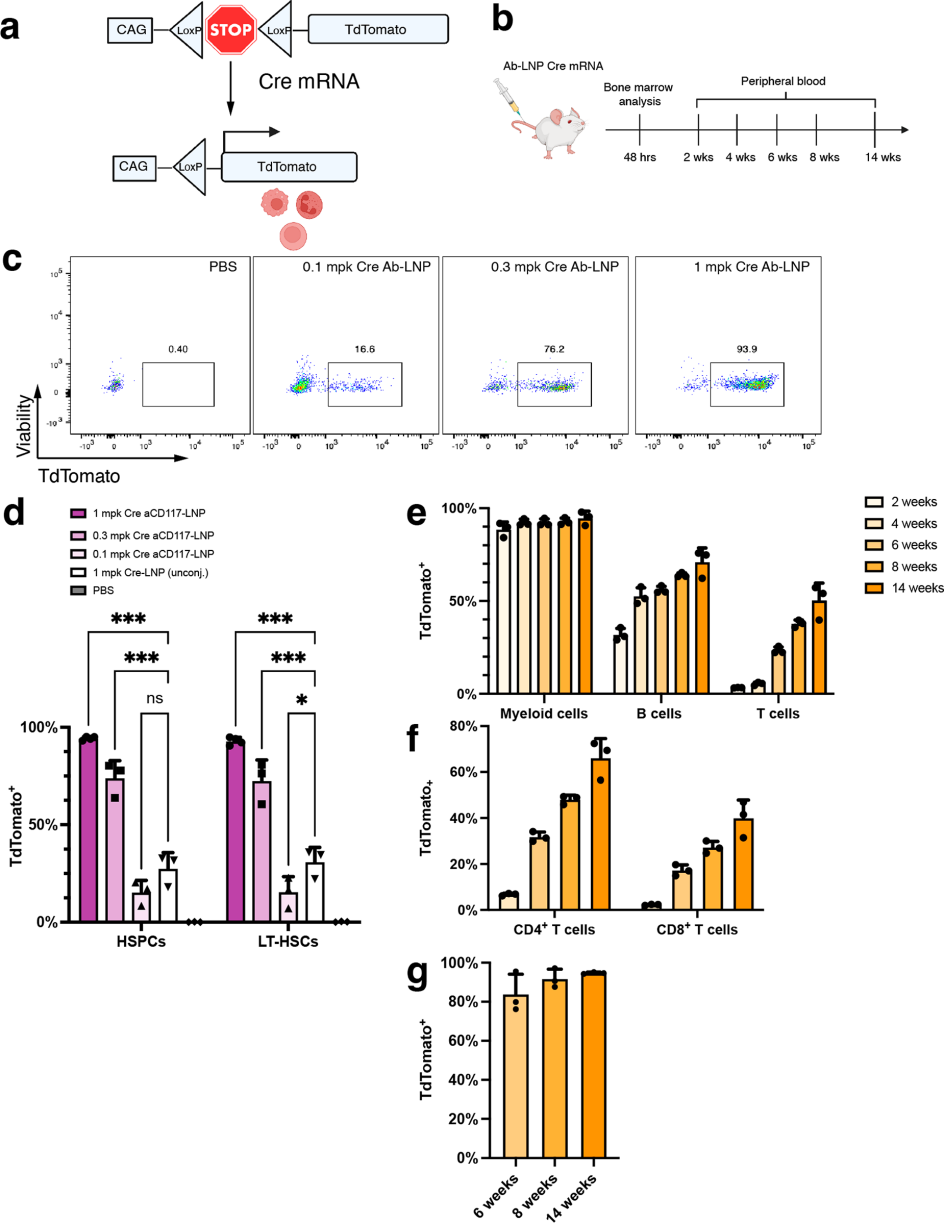
Anti-CD117 LNPs encapsulating Cre mRNA shows high levels of editing *in vivo*. (a) Schematic of the Ai14 transgenic mouse LoxP-flanked stop cassette preventing the transcription of TdTomato. Upon delivery of Cre recombinase *via* Cre mRNA, the stop cassette is excised, and the cell expresses TdTomato. (b) Timeline of experimental workflow for bone marrow and blood analysis. Panels a and b were created with BioRender.com. (c) Representative flow cytometry scatter plots of TdTomato expression in LT-HSCs with varying doses of Cre mRNA. (d) Quantification of the dose–response in both HSPCs and LT-HSCs (*n* = 4 mice for 1 mg kg^–1^Ab-LNP group, *n* = 3 mice for others). Statistics were performed with two-way ANOVA with Tukey’s multiple comparison test. (e) Time course monitoring of TdTomato expression in mature immune cells (*n* = 3 mice). (f) TdTomato expression in T cell subsets (*n* = 3 mice). (g) TdTomato expression in erythrocytes (TER-119^+^) (*n* = 3 mice). Data are represented as mean ± SD (*P < 0.05, **P < 0.01, ***P < 0.001).

We first performed a dose response of anti-CD117 LNP at doses of 0.1, 0.3, and 1 mg kg^–1^ Cre mRNA. At a single administration with a dose of 0.3 mg kg^–1^ (6 μg) mRNA, 48 h after administration, we observed highly efficient delivery with about 75% TdTomato^+^ cells in both the HSPC and LT-HSC populations. When the dose was increased to 20 μg of mRNA, almost all (∼90%) of the HSPCs and LT-HSCs was transfected (Figure 2d). Unconjugated LNPs at a dose of 1 mg kg^–1^ Cre mRNA showed about 25% TdTomato expression, indicating that conventional untargeted LNP formulations have the capability of transfecting HSPCs at a low level. The level of delivery is greatly enhanced by the incorporation of an HSPC targeting ligand. Stemness of the transfected HSPCs was maintained in both short-term and long-term as shown by analysis of the peripheral blood populations (erythrocytes, myeloid cells, B cells, and T cells, gating strategy shown in Figures S6 and S7). At 2 weeks, CD11b^+^ myeloid cells, which consist of granulocytes and monocytes, were already 90% TdTomato^+^. Since B and T cells are cells with longer lifespans, the level of TdTomato expression for those cell types naturally lagged behind that of myeloid cells at 2 weeks (∼32% for B cells and ∼3.3% for T cells), which increased to ∼70% TdTomato^+^ B cells and ∼50% TdTomato^+^ T cells by 14 weeks (Figure 2e). Analysis of T cell subsets (CD4 and CD8) was performed at 4 weeks after LNP administration when the level of TdTomato expression in T cells became more apparent. At each time point, there was a higher population of TdTomato cells in CD4 T cells compared with that in CD8 T cells (Figure 2f). Erythrocytes also showed almost 100% TdTomato expression at 14 weeks (Figure 2g). Overall, almost all the HSPCs were transfected, which then resulted in high levels of corrected progeny in all analyzed immune cell populations.

To analyze the overall biodistribution of anti-CD117 LNPs, we encapsulated firefly luciferase mRNA into LNPs that were conjugated to either anti-CD117 clone 2B8, clone ACK2, or an isotype control (Figure 3a,b). Six hours after injection, luminescence signal for the isotype control LNP was detected primarily in the spleen followed by the liver. In contrast, anti-CD117 LNPs with clone 2B8 showed the highest accumulation in the lung as well as significant accumulation in the bone marrow, intestines, and skin compared to the isotype control. Luminescence signal was also observed in the spleen and liver of antibody conjugated formulations. LNPs conjugated to clone ACK2 also showed lung transfection, albeit at a much lower level, and did not show any difference compared to isotype control within the other tissues. Other than being highly expressed on mast cells, CD117 is expressed on interstitial Cajal cells of the intestine and melanocytes of the skin.^25^ However, CD117 is reported to be expressed at very low levels in the lungs^25,26^ suggesting that the strong luminescence signal in the lungs is a result of nonspecific binding from the antibody. Further work is needed to identify the specific cell type that is being transfected in the lungs and whether there is a specific receptor that is interacting with the antibody. Identification of the cell types and/or receptors can potentially reduce the accumulation in the lung to make our targeted delivery system even more potent and specific to HSPCs.

**Figure 3:**
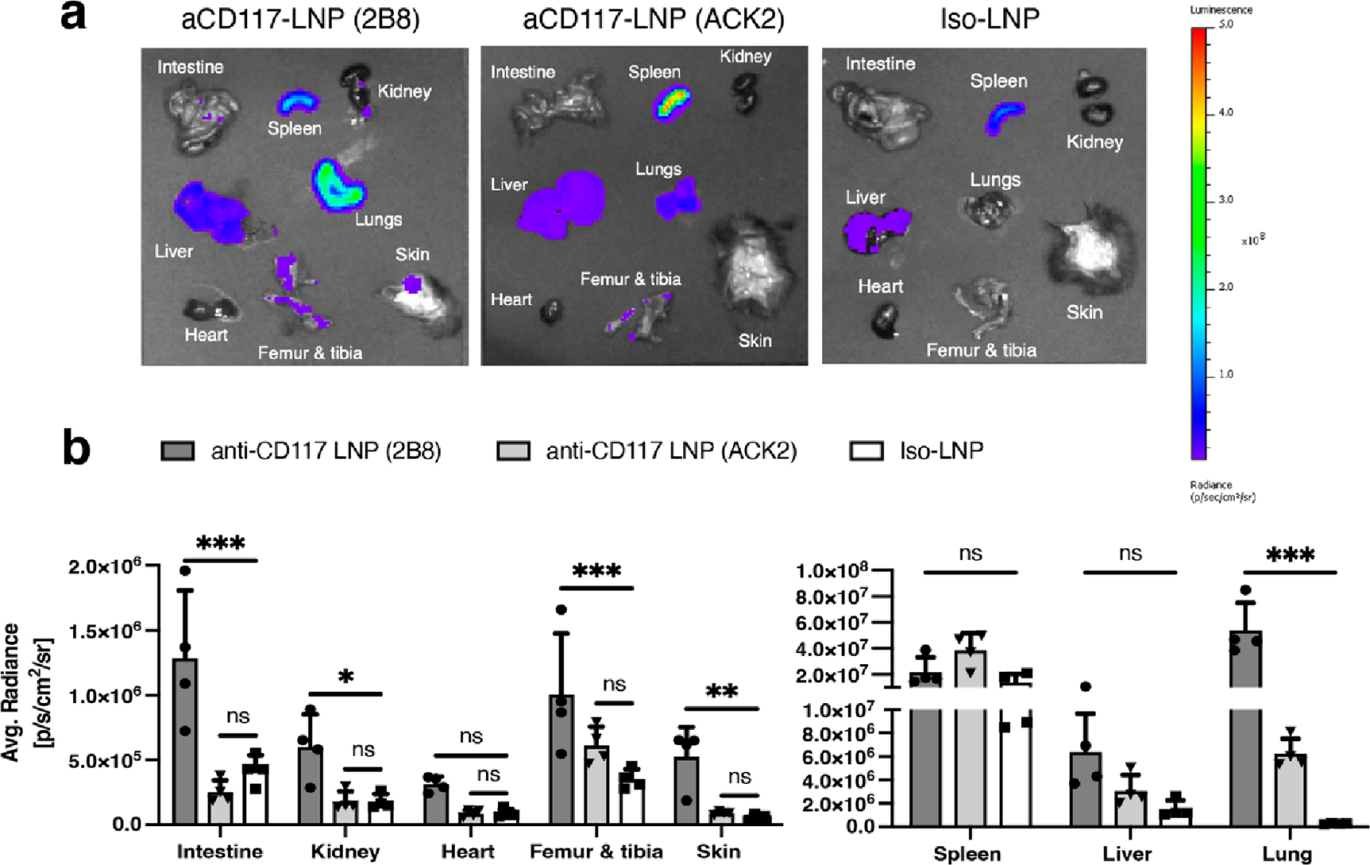
Tissue level biodistribution of LNPs conjugated to anti-CD117 (clone 2B8), anti-CD117 (clone ACK2), or rat IgG2b isotype control. (a) Representative IVIS bioluminescence images of all organs. LNPs containing firefly luciferase mRNA were injected at a dose of 6 μg of mRNA (0.3 mg kg ^–1^). Tissues were collected and imaged 6 h after LNP administration. (b) Quantification of the average radiance. Data represents mean ± SD (*n* = 4 mice). Statistics performed using two-way ANOVA with Dunnett’s multiple comparison test comparing to the isotype control. (*P < 0.05, **P < 0.01, ***P < 0.001).

In summary, *ex vivo* autologous gene therapy has demonstrated strong potential for the treatment of a range of monogenic hematological disorders. However, the process of *ex vivo* gene therapy from stem cell harvest to reinfusion still consists of manufacturing complexities. In addition, there are still many conditioning-associated toxicities especially when higher intensity conditioning is required. Here we developed an **in vivo** RNA delivery system to HSPCs using an LNP conjugated to an antibody against CD117. Importantly, we identified several key formulation requirements, including (1) that different antibody clones against the same receptor had markedly different levels of LNP delivery **in vivo** (clone 2B8 achieved 85% uptake, while clone ACK2 had minimal uptake) and (2) longer alkyl chain PEG-lipid (C18) greatly improved the delivery efficacy of systemically administered nanoparticles to the bone marrow. The optimized formulation showed the capability of delivering both small RNA (siCD45) and longer mRNA (Cre mRNA) **in vivo**. Interestingly, CD117 targeting could be achieved with many different ionizable lipid formulations.

In a mouse model for gene editing, anti-CD117 LNPs encapsulated with Cre recombinase mRNA were capable of editing almost all HSPCs within the bone marrow *in vivo*. Importantly, this included editing of over 90% of LT-HSPCs. We believe the formulation described here shows the potential of antibody targeted LNPs to deliver RNA to HSPCs and provides a framework for the generation of other antibody targeted LNPs.

## Supporting Information Available

Materials and methods, size exclusion chromatography profile of Ab-LNPs, representative flow cytometry gating, and supplementary figures (PDF)
