# Supplementary material for: In Vivo RNA Delivery to Hematopoietic Stem and Progenitor Cells via Targeted Lipid Nanoparticles
Source: Nano Lett. 2023 Mar 29;23(7):2938–44. doi: 10.1021/acs.nanolett.3c00304 (PMC10103292; doi:10.1021/acs.nanolett.3c00304)
Supplement: Supplementary file 1 — nl3c00304_si_001.pdf [file nl3c00304_si_001.pdf]

In vivo RNA delivery to hematopoietic stem and progenitor cells via targeted lipid nanoparticles

Dennis Shi[1,2], Sho Toyonaga[2,3], Daniel G. Anderson[1,2,4,5,]*

[1] Department of Chemical Engineering, Massachusetts Institute of Technology, Cambridge, MA 02139, USA.

[2] David H. Koch Institute for Integrative Cancer Research, Massachusetts Institute of Technology, Cambridge, MA 02139, USA.

[3] FUJIFILM Pharmaceuticals U.S.A., Inc., Cambridge, MA 02141, USA.

[4] Harvard-Massachusetts Institute of Technology, Division of Health Science and Technology, Massachusetts Institute of Technology, Cambridge, MA 02139, USA.

[5] Institute for Medical Engineering and Science, Massachusetts Institute of Technology, Cambridge, MA 02139, USA.

*Address correspondence to dgander@mit.edu

Supporting Information

Methods

Materials. Ionizable lipids (MC3, Lipid 5, SM-102, and ALC-0315) were purchased from Cayman Chemicals. cKK-E12 was synthesized as previously described[1]. DSPC, DMG-PEG2000, DSG-PEG2000 were purchased from Avanti Polar Lipids. Cholesterol was purchased from Sigma-Aldrich. DPG-PEG2000, DSPE-PEG2000-Maleimide was purchased from NOF America. Antibodies for conjugation (anti-CD117 clone 2B8, anti-CD117 clone ACK2, rat IgG2b isotype control, anti-CD44, anti-CD49, anti-IL-6R) were purchased from Bio X Cell.

Nanoparticle formulation. Lipid nanoparticles were prepared via microfluidic mixing as previously described[2]. Briefly, an ethanol phase was prepared consisting of ionizable lipid, DSPC, cholesterol, PEG-lipid, and functionalized maleimide PEG-lipid at a ratio of 50:10:38:1.5:0.5 and then rapidly mixed with an aqueous solution of RNA in 10 mM citrate buffer pH 3.0 at an aqueous to ethanol volume ratio of 3:1. For *in vivo* uptake experiments, a lipid dye DiR (ThermoFisher) was included in the ethanol phase at 0.5 mol% for direct labeling of the nanoparticles. After formulation, LNPs were dialyzed using Slide-A-Lyzer 10K MWCO dialysis devices (ThermoFisher) for at least 18 hours at 4°C in PBS containing 10 mM EDTA. For characterization, nanoparticle size and PDI were determined with Malvern ZetaSizer and RNA encapsulation efficiency was determined via Quant-IT RiboGreen (ThermoFisher) assay.

Antibody conjugation. LNPs containing DSPE-PEG2000-Maleimide were dialyzed at 4°C overnight in PBS with 10 mM EDTA. To prepare antibodies for conjugation, antibodies were first reduced with 5 eq. of TCEP (5 mM stock solution in PBS, Sigma-Aldrich) at 37°C for 1 hr with gentle shaking. Excess TCEP was removed by passing antibody solution through a Zeba 7K

MWCO desalting column (ThermoFisher). Concentration of reduced antibody was quantified via absorbance at 280 nm via NanoDrop (ThermoFisher). Antibody was then incubated with functionalized LNPs for one hour at room temperature with end-over-end mixing and then stored at 4°C prior to purification. Ab-LNPs were purified with size exclusion chromatography using bench-top qEVoriginal columns (Izon Sciences) with PBS as the mobile phase. Fractions containing Ab-LNPs were combined and then concentrated using Amicon ultracentrifugation filters (Sigma-Aldrich) and then stored at 4°C prior to injection. Protein concentration was determined by BCA assay (ThermoFisher).

Cell culture and qPCR. EML cells (ATCC CRL-11691) were cultured in IMDM (ATCC) supplemented with 1% Penicillin/Streptomycin (Gibco), 20% HI-FBS (Gibco), and 200 ng/µL of recombinant murine stem cell factor (Peprotech). Cells were maintained at 37°C and 5% CO_2. For *in vitro* transfection, EML cells were plated at a density of 40,000 cells per well of a 96-well U-bottom plate. Then 50 ng of siCD45 LNP was added to the well. 48 hours after transfection, RNA level silencing was measured by using qPCR. For isolation of total RNA, cells were washed with PBS and then spun down and resuspended with 50 µL QuickExtract RNA solution (Lucigen). 2 µL of the QuickExtract lysate was used directly with Luna Universal One-Step qPCR (New England Biolabs) using TaqMan probes (ThermoFisher). Percent knockdown was quantified with the ddCt method using B2m as the housekeeping gene and compared to untreated PBS control.

Animal studies. All animal study protocols were approved by the MIT Committee on Animal Care (CAC) and supervised by MIT Division of Comparative Medicine veterinary staff. C57BL/6 (F, 6 to 10 weeks) mice were purchased from Jackson Labs (#000664). B6.Cg-$^{Gt(ROSA)26Sortm14(CAG-tdTomato)Hze}$/J, Ai14, mice (purchased from Jackson Labs, #007914) were bred in

house and both males and females were used for experiments. Mice were housed in MIT animal facility.

Cell isolation for flow cytometry staining. To isolate bone marrow cells, mice were sacrificed and then the skin and muscle was first removed from the right hind leg (femur and tibia). Then the femur and tibia were cut in half and bone marrow cells were collected via centrifugation as previously described[3]. After centrifugation, the cell pellet was resuspended in PBS and passed through a 70-µm strainer. Cells were then spun down at 450 x g for 5 minutes and resuspended with 1 mL RBC Lysis Buffer (Qiagen) and incubated on ice with gentle shaking for 10-15 minutes. RBC lysis was quenched by addition of excess PBS and then cells were spun down and resuspended in FACS buffer (PBS with 1% BSA and 2.5 mM EDTA) for antibody staining. Cells were stained with fixable viability dye (ThermoFisher) for 30 minutes on ice protected from light. After viability staining, cells were blocked with TruStain FcX PLUS (BioLegend) for 10 minutes on ice and then incubated with antibody solution for 30 minutes on ice. Antibodies used were FITC-CD3e, FITC-TER-119, FITC Gr-1, FITC B220, FITC CD11b, BV605 CD117, PE Sca1, PE/Cyanine7 CD34, APC CD135, BV421 CD45.2. For TdTomato experiments, BV785 Sca1 was used instead of PE. After staining, cells were washed 2x with PBS and then fixed with 4% formaldehyde in PBS.

For staining of mouse whole blood, blood was collected via submandibular bleed into EDTA coated BD Microtainer (BD Biosciences). Then 100 µL of whole blood was lysed with 1 mL RBC Lysis Buffer (Qiagen) and incubated on ice with gentle shaking for 10 minutes. Cells were spun down at 450 x g to remove lysis buffer and washed once with 1 mL of PBS. After washing, cells were resuspended in staining buffer and incubated with immune cell markers (Alexa Fluor 700-CD11b, FITC-CD3e, PE/Cyanine7-CD4, APC-CD8, BV421-CD45.2, and BV605-

CD45R/B220). For analysis of erythrocytes, 1 μL of unlysed whole blood was added to 100 μL FACS staining buffer and then stained with viability dye and FITC TER-119 (BioLegend). After staining, cells were analyzed on an LSR Fortessa (BD Biosciences), and data was analyzed with FlowJo.

In vivo CD45 knockdown. To assess silencing of CD45 *in vivo*, C57BL/6 mice (F, 6-10 weeks) were injected with varying doses of siCD45 LNPs via tail vein. 72 hours after administration, mice were sacrificed, and the right hind leg was dissected and then kept on ice prior to processing and flow cytometry staining. Knockdown was assessed by comparing the median fluorescence intensity of CD45 of treated LSK cells to the MFI of untreated control.

In vivo luciferase biodistribution. To assess biodistribution of Ab-LNPs, C57BL/6 mice (F, 6-10 weeks) were injected with 0.3 mg/kg of luciferase mRNA LNPs via tail vein. 6 hours after administration, mice were injected i.p. with 130 μL of a 30 mg/mL D-Luciferin (PerkinElmer) solution in PBS. Ten minutes after luciferin administration, mice were sacrificed, and major organs (lung, heart, liver, spleen, intestines, and femur & tibia, skin) were collected and imaged on an IVIS Spectrum imaging system (PerkinElmer) and luminescence was quantified with LivingImage software (PerkinElmer).

In vivo Cre delivery. Ai14 mice (6-10 weeks) were injected via tail vein with Cre mRNA Ab-LNPs. For analysis of bone marrow HSPCs, mice were sacrificed after 48 hours and then femur and tibia were processed for flow cytometry staining. For immune cell lineage tracing, 100 μL of whole blood was collected every two to three weeks and then stained for flow cytometry according to the protocol above.

Statistics. Means were compared with either one-way ANOVA or two-way ANOVA for comparison between multiple groups. Dunnett's multiple comparison test was used when comparing to a control group while Tukey's multiple comparison test was used when comparing every mean to every other mean. For comparison between two groups, unpaired two-tailed T-test was used. All statistics were performed with GraphPad Prism 9.

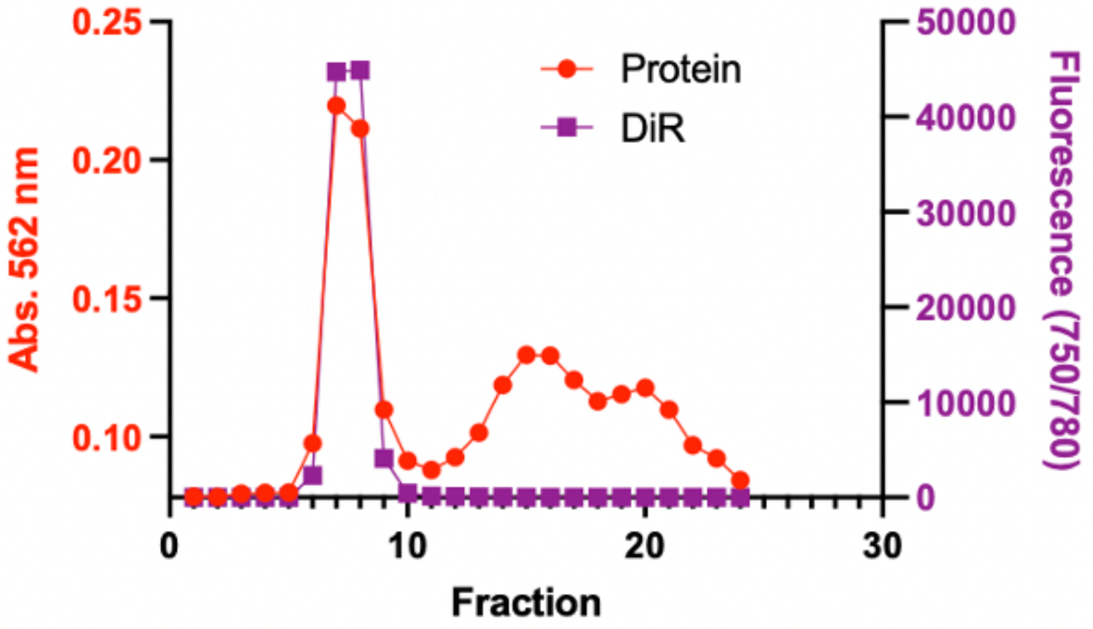

Figure S1. Elution profile of Ab-LNP from size exclusion chromatography. 0.5 mL fractions were collected. Fractions containing antibody were determined via protein BCA assay (read at absorbance of 562 nm) while fractions containing DiR fluorescently labeled LNP were determined via reading the fluorescence intensity of at Ex. 750 and Em. 780. The overlap of the two peaks in fractions 6-9 corresponds to successfully conjugated Ab-LNPs.

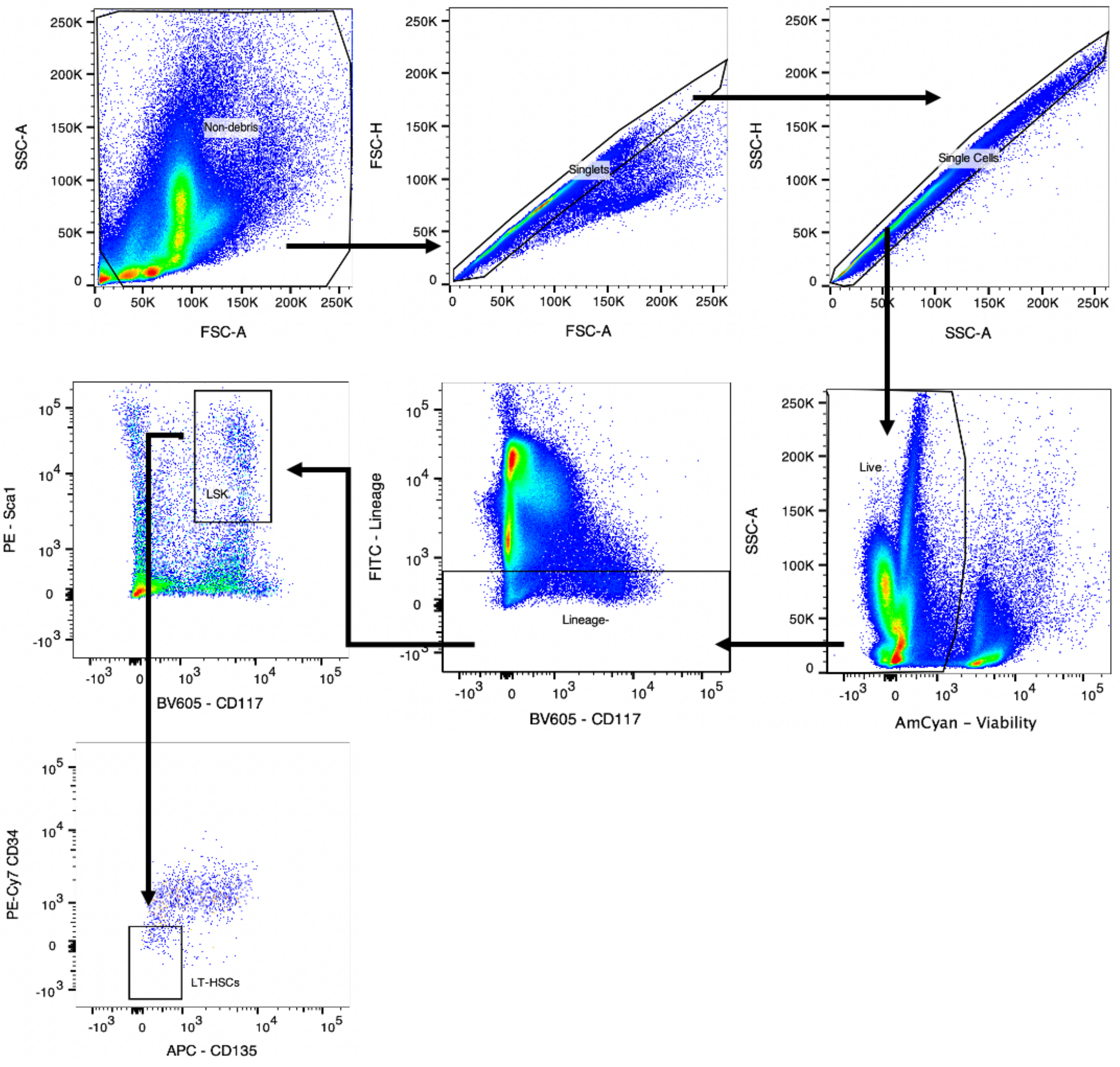

Figure S2. Representative gating of bone marrow HSPCs and LT-HSCs. Cells are gated based on singlets and live cells (Aqua viability dye) followed by gating for FITC lineage events and double positive for PE-Sca1 and BV605-CD117. LT-HSCs are further defined as double negative for APC-CD135 and PE-Cy7-CD34. For uptake experiments this LSK population is analyzed by DiR signal and for knockdown experiments this population is analyzed with the MFI for CD45.

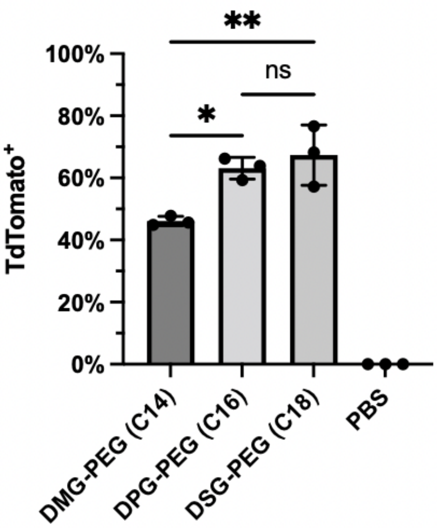

Figure S3. Effect on PEG-lipid alkyl length on mRNA delivery to HSPCs in the bone marrow. Ab-LNP formulations containing Cre mRNA and different PEG-lipids were injected to Ai14 mice at a dose of 0.3 mg kg^{-1}. 48 hours after injection, mice were sacrificed and right hind leg was collected for flow cytometry analysis. TdTomato expression was evaluated in bone marrow LSK cells. Statistics performed by one-way ANOVA with Tukey's multiple comparison test (*$P < 0.05$, **$P < 0.01$).

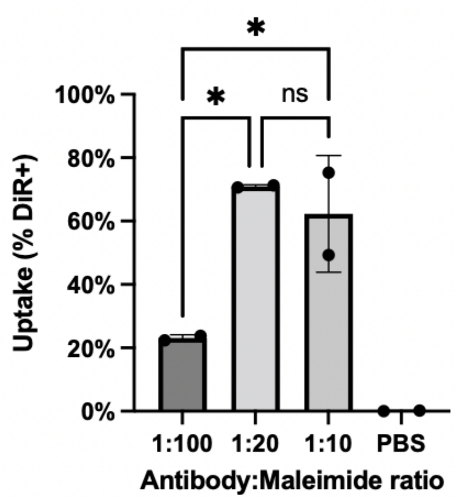

Figure S4. Optimum ligand density of Ab-LNPs. The ligand density was adjusted by titration of the amount of antibody added to the reaction mixture. These formulations were then injected intravenously at a dose of 0.3 mg kg^{-1} siCD45. Bone marrow cells were collected 72 hours after administration and analyzed for DiR fluorescence signal in BM HSPCs (n = 2 mice). Statistics performed by one-way ANOVA with Tukey's multiple comparison test. (*$P < 0.05$).

**Sample**	**Z-Average (nm)**	**PDI**	**EE%**
Unconjugated LNP	71.24	0.06	90
aCD117 LNP	91.04	0.14	92

Table S1. Nanoparticle physicochemical characterization before and after conjugation.

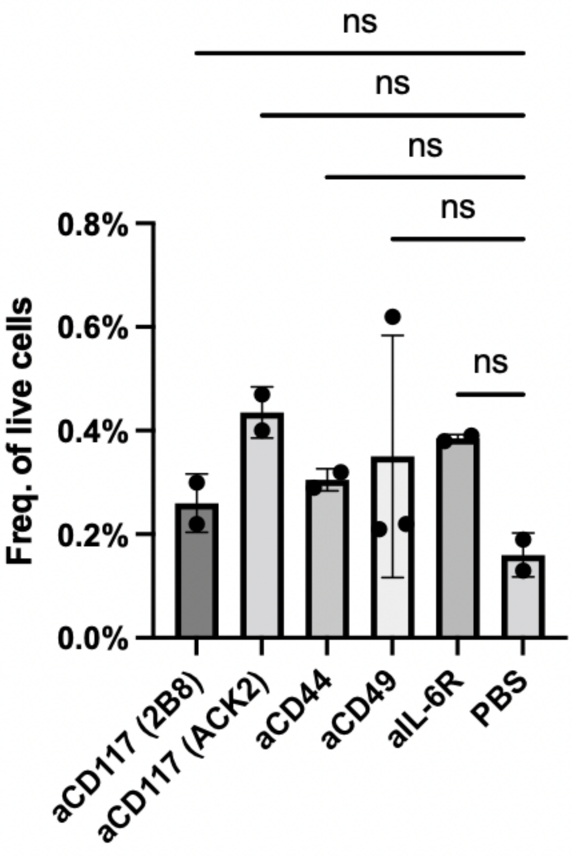

Figure S5. Treatment of ligand decorated LNPs at a dose of 1 mg kg^{-1} siCD45 does not result in any depletion or clearance of HSCs. Statistics determined by one-way ANOVA with Dunnett's multiple comparison to the PBS control group (n = 3 mice for anti-CD49 group, n = 2 mice for other groups). Significance was defined as $P < 0.05$.

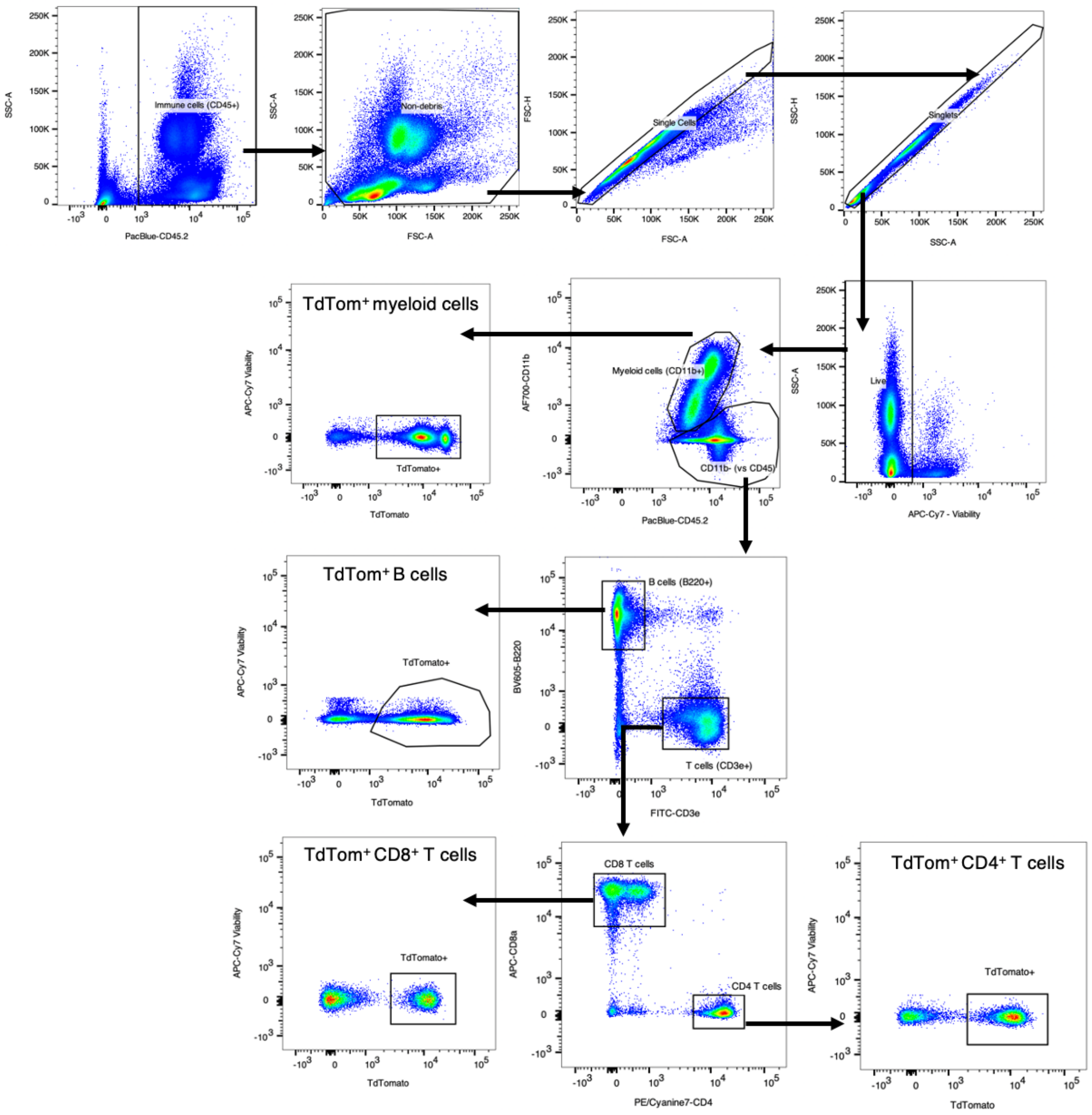

Figure S6. Representative gating strategy for analysis of TdTomato peripheral blood populations at 14 weeks. Myeloid cells were gated based on $CD45^+$ $CD11b^+$. B cells were gated based on $CD45^+$ $CD11b^-$ $B220^+$. T cells were gated based on $CD45^+$ $CD11b^-$ $CD3e^+$ (n = 3 mice).

.

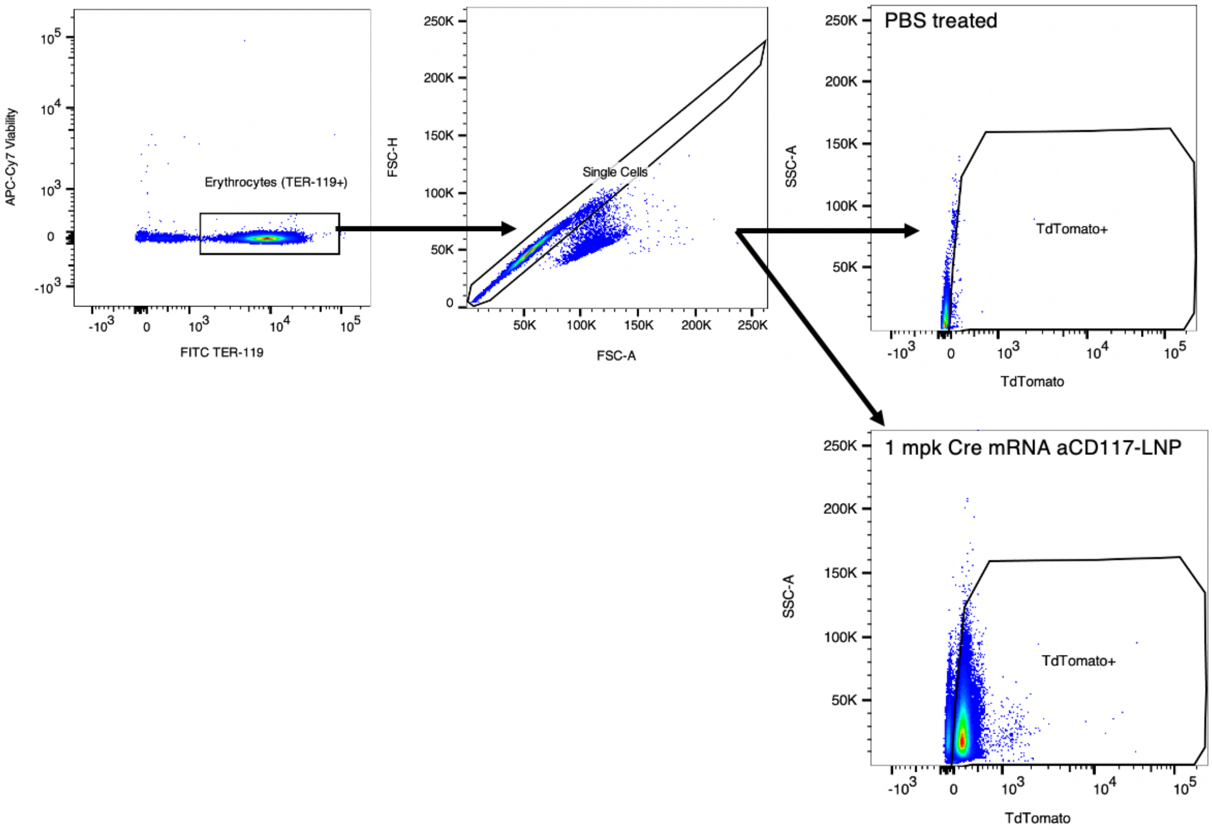

Figure S7. Representative gating strategy for analysis of TdTomato erythrocytes at 14 weeks (n = 3 mice). Erythrocytes were gated based on live TER-119^{+} cells. TdTomato gate was drawn based on FMO controls.

References

1. Dong, Y. *et al.* Lipopeptide nanoparticles for potent and selective siRNA delivery in rodents and nonhuman primates. *Proc. Natl. Acad. Sci. U.S.A.* **111**, 3955–3960 (2014).

2. Chen, D. *et al.* Rapid discovery of potent siRNA-containing lipid nanoparticles enabled by controlled microfluidic formulation. *J. Am. Chem. Soc.* **134**, 6948–6951 (2012).

3. Amend, S. R., Valkenburg, K. C. & Pienta, K. J. Murine hind limb long bone dissection and bone marrow isolation. *J. Vis. Exp*. **110**, (2016).
